# Dietary patterns and mental health after myocardial infarction

**DOI:** 10.1371/journal.pone.0186368

**Published:** 2017-10-16

**Authors:** Nathaly Rius-Ottenheim, Daan Kromhout, Femke P. C. Sijtsma, Johanna M. Geleijnse, Erik J. Giltay

**Affiliations:** 1 Leiden University Medical Centre, Department of Psychiatry, Leiden, the Netherlands; 2 Spaarne Gasthuis Hospital, Department of Psychiatry, Haarlem, the Netherlands; 3 Division of Human Nutrition, Wageningen University, Wageningen, the Netherlands; 4 Department of Epidemiology, University Medical Centre, University of Groningen, Groningen, the Netherlands; TNO, NETHERLANDS

## Abstract

**Background:**

Diet has been associated with better mental health in general populations, but less is known on this association in patients with a history of coronary heart disease.

The objective of this study is to examine the cross-sectional associations between dietary patterns and mental health in elderly patients with a history of myocardial infarction.

**Methods:**

Data were drawn from the final assessment of the Alpha Omega cohort that monitored patients with a history of myocardial infarction (age range 60–80 years). 2171 patients with complete data for diet and mental health were included in this study. Diet was assessed with the 203-item Food Frequency Questionnaire, and subsequently categorized into two scores: the Dutch Healthy Nutrient and Food Score (DHNaFS) and the Dutch Undesirable Nutrient and Food Score (DUNaFS). Depressive symptoms, assessed with the Geriatric Depression Scale (GDS-15), and dispositional optimism, assessed with the 4-item questionnaire (4Q), were cross-sectionally analyzed in relation to dietary patterns using linear regression analysis.

**Results:**

Patients were on average 72.2 years old and 79.5% were male. The DHNaFS score was associated with less depressive symptoms and higher dispositional optimism (β = -0.108; P<0.001; and β = 0.074; P<0.001), whereas no associations were found with the DUNaFS score. Particularly, consumption of vegetables, fruits, whole grains, fish, and low fat-dairy were associated with less depressive symptoms and higher optimism. Similar associations were found when analyzing the association between average DHNaFS score over the preceding 41 months with depression β = -0.085; P<0.001) and higher dispositional optimism (β = 0.084; P<0.001).

**Conclusions:**

A healthy dietary pattern, in particular a higher consumption of vegetables, fruit, whole grains, fish and low-fat dairy, was associated with less depressive symptoms and higher optimism. However, given the cross-sectional nature of our analyses, our findings may also be explained by more optimistic participants making healthier food choices. Therefore, future prospective or interventions studies are needed to establish the direction of causality of this association.

**Trial registration:**

ClinicalTrials.gov NCT03192410.

## Introduction

Depression is a highly prevalent mental disorder among patients with a history of myocardial infarction (MI) [1, 2] and is associated with a decreased quality of life and high costs in healthcare use [3]. Moreover, comorbid depression is related to worse cardiovascular outcomes and increased mortality [4, 5]. Thus far, conventional depression treatments such as pharmacotherapy and psychotherapy have only yielded modest results [6–8], and subclinical depressive symptoms often persist and remain untreated [9]. Research is therefore needed to identify alternative or additional lifestyle factors that are associated with mental health in cardiac patients.

Diet may be such a modifiable risk factor. Systematic reviews in general populations have shown a beneficial effect of healthy dietary patterns that include high consumption of fruits, vegetables, fish, olive oil, nuts, and legumes, and an adverse effect of unhealthy dietary patterns that include high consumption of processed food, sugar and fat [10–13]. In line with these reviews, later observational studies have reported an inverse association between healthy dietary patterns and depressive symptoms [14–17]. Also, a large randomized trial (the PREDIMED study) showed that a low fat, Mediterranean diet decreased the incidence of depression in a population at high cardiovascular risk [18]. Their results suggested an association between a dietary pattern enriched with extra-virgin olive oil and mixed nuts, but only in a sensitivity analysis among the participants with diabetes mellitus type 2.

Dietary patterns have not only been related to the state-like depressive symptoms, but also to positive psychological traits [19]. Dispositional optimism, a positive trait that refers to an individual’s generalized positive expectancies towards the future [20], has been associated with healthier dietary patterns in younger adults [21, 22], older men [23], and post-menopausal women [24]. More recently, a cross-sectional study among Spanish women with fibromyalgia, reported an association between optimism and higher consumption of fruit, vegetables, and fish [25]. Most of these previous studies used short questionnaires to estimate dietary habits [22, 25], while one did use a standardized dietary history method [23]. Therefore, more studies on the association between dietary patterns and dispositional optimism are needed.

With the present study, we aimed to assess the associations of dietary patterns with depressive symptoms and optimism in cardiac patients, using detailed dietary data. We hypothesized that a healthy nutrient-rich food score would be associated with lower depressive symptoms and higher optimism; whilst food scores relatively high in solid fats, sodium and/or added sugar would be associated with a poorer mental health.

## Methods

### Study sample

This analysis was carried out among participants of the final wave of the Alpha Omega Trial (AOT) (ClinicalTrials.gov no. NCT00127452). The design of this trial has been described in detail elsewhere [26]. Briefly, the AOT was a randomized, double-blind controlled trial that investigated the effects of n-3 fatty acids supplementation on relapse of cardiovascular events in a sample of patients with a history of MI. These post-MI patients were recruited by cardiologists from 32 hospitals between 2002 and 2006, and the date of last follow-up was December 23rd, 2009. Subjects were considered eligible if they were aged between 60 to 80 years at baseline, had a verified diagnosed MI up to 10 years before randomization, were not cognitively impaired (i.e. MMSE score ≥ 22 points), and were willing to provide written informed consent [26, 27]. These selection criteria resulted in a sample of 4837 eligible subjects at baseline (T1) who were subsequently randomized into four groups of the n-3 fatty acids. Of this initial cohort, 370 participants died during follow-up. Results of this primary analysis on the association between diet and cardiovascular outcomes have already been published elsewhere [26]. For the current secondary analysis, we focused on data from the final wave as mental health was only assessed at this time point (T2). At the final wave, owing to financial constraints, dietary data were only available for the first 2675 patients who were randomized until August 2006, yielding 55% of the initial cohort. Of these remaining 2675 participants, 293 participants were excluded because they had more than one missing on the diet assessment, 46 participants because they had more than one missing on the mental health assessment, and 165 participants because they had more than one missing on both the mental health assessment and the diet assessment. Thus, 2171 participants with complete data were included in our cross-sectional analyses on the association between diet and mental health.

The study was conducted in compliance with the Declaration of Helsinki and approved by a central medical ethics committee (Haga Hospital, Leyenburg, The Hague, Netherlands) and by the ethics committee at each participating hospital Written informed consent was obtained from all participants.

### Dietary data

Dietary data were collected by a 203-item food frequency questionnaire (FFQ) developed for the Alpha Omega Trial. The FFQ was an extended and adapted (for people aged 60–80 years) version of a reproducible and biomarker validated FFQ [28, 29]. Patients were asked to report the usual intake of foods consumed during the previous month; questions on the frequency, amount, and type of foods and preparation methods were included. Trained dieticians checked the returned questionnaires and obtained additional information on unclear or missing items by phone. Quality assurance procedures included double entry of all FFQ data. The food data were converted into energy and nutrient intake by using the 2006 Dutch food composition database [30]. The 203 food items were collapsed into 24 food groups according to criteria derived from the Guidelines Food Choice of the Netherlands Nutrition Center.

The calculation of the food scores within the AOT has been previously reported in more detail [31]. We made a distinction between food groups consisting of nutrient-dense foods that contribute importantly to the nutrient supply and are typical for the Dutch diet and food groups that are relatively high in solid fats (saturated and trans fats) and/or sodium and/or added sugar. To create two food scores, the food groups were categorized into quintiles of consumption, with study patients receiving a score of 0 to 4 for each of the 24 food groups, and then summed. The Dutch Healthy Nutrient and Food Score (DHNaFS) included 11 nutrient-dense food groups: vegetables, fruit, whole grains, protein-rich plant foods (i.e. legumes), potatoes, lean meat, fish, eggs, low-fat milk and yogurt, oils and soft margarines, and non-caloric drinks. The Dutch Undesirable Nutrient and Food Score (DUNaFS) included 13 food groups that were high in solid fats, sodium, and/or added sugar: processed food, high-fat meat, processed meat, full-fat milk, cheese, refined grains, butter and margarines, soups, spreads, ready-to-eat meals, sweet snacks, and sugar-sweetened beverages.

### Mental health assessment

Depressive symptoms were assessed with the 15-item version of the Geriatric Depression Scale (GDS-15). The GDS-15 is a validated self-report instrument designed to assess depressive symptoms in older people [32]. This instrument is especially useful to measure depressive symptoms in older patients with coronary heart disease, as it excludes somatic symptoms that might also be related to physical illnesses rather than to depressive symptoms [33]. The total score ranges from 0 to 15 points, with higher scores indicating more depressive symptoms. For the computation of the GDS-15 score, two missing items were allowed, being subsequently imputed with the mean of the remaining items. Furthermore, a (familiar) history of depression and use of antidepressants were reported.

Dispositional optimism was assessed using the four-item questionnaire (4Q) [34] from a survey of Statistics Netherlands (CBS) consisting of the following statements–“I still expect much from life”, “I do not look forward to what lies ahead for me in the years to come”, “My days seem to be passing by slowly”, and “I am still full of plans” (our translations). Subjects were asked to express the extent of their agreement with each of the items, coding their responses on a 0–2 Likert type scale. Within the four score items, two negatively stated items required reversed coding. The additional answer category “do not know” was also coded as the midpoint (score 1). The optimism questionnaire scores range from 0–8 points, with higher scores being indicative of higher optimism levels.

### Covariates

At T2, data were collected on demographic factors, lifestyle, medical history, current health status, and medication use. Information about the highest attained level of education was used as an indicator of socioeconomic status, with more than 11 years of education as the upper category. Marital status was dichotomized into the categories being married (or cohabiting) or not (i.e. unmarried, divorced, widow). Smoking habits were categorized into never smoked, smoked in the past, and current smoking. Alcohol use was categorized into no alcohol use, use of 1 unit per week, and use of more than 1 alcohol units per week. Body mass index (BMI) was calculated from the measured weight and height. Reported self-rated health was dichotomized into excellent to good self-rated health and moderate to poor self-rated health. Physical activity was assessed by the validated Physical Activity Scale for the Elderly (PASE) questionnaire and categorized as no activity or only light activity, <30 min/day moderate to vigorous activity, and ≥30 min/day moderate to vigorous activity [35]. Self-reported medication of the patients was coded according to the Anatomical Therapeutic Chemical Classification System (ATC). ATC codes for antidepressant medication were N06A and N06AX.

### Statistical analyses

Sociodemographic and baseline characteristics were summarized using descriptive statistics. Categorical variables were presented as proportions across quintiles of both food scores. Continuous data were presented as mean and standard deviation. As the variable depressive symptoms was positively skewed and optimism was negatively skewed, these variables are presented as median and interquartile ranges. We additionally performed multiple imputation to evaluate the effects of missing data and avoid the potential bias and decreased statistical power associated with complete case analysis. The imputation model incorporated three key variables used in the analysis and fourteen auxiliary (confounding) variables. The results of five imputed data-sets were compared with the complete case analyses. The Multivariate Imputation by Chained Equations approach was used as a multiple imputation method.

An analysis of covariance and linear regression were conducted to explore the associations of the food scores with depressive symptoms, on the one hand, and dispositional optimism, on the other hand. We used the standardized beta-coefficients as estimates of effect size over the increasing quintiles. Associations were first assessed in a crude model, using a Jonckheere-Terpstra non-parametric test. Subsequently, we repeated the analyses adjusting first for age and sex (Model 1) and additionally for education, marital status, physical activity, body mass index, high alcohol use, current smoking, use of antidepressants, family history of depression, self-rated health, chronic disease, and treatment group (Model 2). Furthermore, linear regression analyses were performed to evaluate the association of individual foods of the healthy composite food score with depressive symptoms, on the one hand, and dispositional optimism, on the other hand.

To assess the association with longer term dietary patterns, analyses were repeated with the average food scores (at T1 and T2) as the independent variable and depressive symptoms and dispositional optimism as the dependent variables. In a sensitivity analysis, the same analyses were conducted in the subgroup of patients not using antidepressants at T1 and T2.

All tests were two-tailed with *p*<0.05 denoting statistically significance. The software used was SPSS version 24.0 (SPSS Inc., Chicago, Ill).

## Results

### Study population

The included 2171 patients had a mean age of 72.2 years and were predominantly male (79.5%). Most participants were married and had a low educational level. The mean BMI was 27.7 kg/m^2^ and 20.5% of the patients was physically active. Alcohol consumption was predominantly moderate and most of the patients were former smokers. Patients rated their own health predominantly as good (72.5%). Regarding mental health, the median GDS-15 score was 1.00 points, whereas the median score for the 4Q-optimism scale was 6.67 points. Only 4.7% reported use of antidepressants and 19.6% had a positive family history for mood disorders.

Compared to the included patients, excluded participants were more likely to be older, unmarried, being unhealthier (i.e., more smoking, higher BMI, and more chronic disease) men, and therefore more likely to rate their health as poor and scoring slightly higher on the depression scale. The results of the multiple imputation analysis showed a pooled beta of -0.120 (t = -4.34; p = 0.004) for depression scores in relation to healthy diet and a pooled beta of 0.08 (t = 2.70; p = 0.03) for optimism scores in relation to healthy diet. Similar effect estimates were found after multiple imputation of the missing data, suggesting that findings were robust.

### Cross-sectional findings

Table 1 shows the baseline characteristics across quintiles of the DHNaFS and the DUNaFS scores. Patients in the highest quintile of the healthy food score were more likely to be men, be married, and had higher education levels. They were also more physically active, were less likely to smoke, and subjectively rated their health as better than patients in the lowest quintile. Alcohol consumption, on the contrary, tended to be higher in the highest quintile of the DHNaFS score. Differences for lifestyle variables were smaller across quintiles of the DUNaFS score.

**Table 1:** Characteristics of 2171 post-myocardial infarction patients at T2 depending on DHNaFS and DUNaFS quintiles.

	Dutch Healthy Nutrient and Food Score (DHNaFS)			Dutch Undesirable Nutrient and Food Score (DUNaFS)		
	Q1	Q3	Q5	Q1	Q3	Q5
Age, years mean (SD)	72.9 (5.5)	72.4 (5.4)	71.7 (5.2)	72.7 (5.4)	72.1 (5.6)	71.9 (5.3)
Men. No. (%)	295 (72.8%)	422 (79.2%)	399 (86.9%)	273 (66.1%)	274 (79.2%)	358 (90.9%)
Higher education^a^. No (%)	31 (7.7%)	86 (16.2%)	94 (20.6%)	41 (10.0%)	58 (16.9%)	70 (17.9%)
Married. No (%)	286 (70.6%)	432 (81.2%)	395 (86.1%)	305 (74.0%)	289 (83.5%)	334 (84.8%)
Body mass index. mean (SD)	27.7 (3.9)	27.6 (3.3)	27.7 (3.6)	28.1 (3.9)	27.7 (3.8)	27.3 (3.6)
Physically active. No. (%)	46 (11.4%)	120 (22.6%)	121 (26.4%)	83 (20.4%)	58 (16.9%)	94 (23.9%)
Current Smoker. No. (%)	76 (18.7%)	72 (13.5%)	44 (9.6%)	70 (16.9%)	48 (13.9%)	46 (11.7%)
Alcohol use. No (%)						
• >1 glass a week	269 (66.7%)	381 (71.8%)	363 (79.1%)	291 (70.6%)	248 (72.3%)	294 (74.6%)
• <1 glass a week	16 (4.0%)	46 (8.7%)	25 (5.4%)	18 (4.4%)	25 (7.3%)	27 (6.9%)
• No use	118 (29.3%)	104 (19.6%)	71 (15.5%)	103 (25.0%)	70 (20.4%)	73 (18.5%)
Poor self-rated health. No (%)	146 (36.1%)	144 (27.1%)	102 (22.2%)	123 (29.9%)	95 (27.5%)	92 (23.4%)
Chronic disease. No (%)	138 (34.1%)	171 (32.1%)	136 (29.6%)	159 (38.5%)	94 (27.2%)	107 (27.2%)
Antidepressant use. No (%)	26 (6.4%)	26 (4.9%)	14 (3.1%)	29 (7.0%)	14 (4.0%)	11 (2.8%)
Family history of depression. No (%)	56 (13.9%)	98 (18.5%)	117 (25.5%)	79 (19.3%)	70 (20.3%)	84 (21.4%)
Depressive symptoms GDS, median (IQR)	2 (3)	1 (2)	1 (2.1)	2 (3)	1 (2.1)	1 (3)
Optimism 4Q, median (IQR)	6 (2)	7 (2)	7 (2)	6 (3)	7 (2)	7 (2.7)

Participants were included according to completeness of data for all variables.

Higher education is defined as having more than 11 years of education or having at least completed secondary education.

Body mass index is calculated as weight in kilograms divided by height in meters squared.

Chronic disease is defined as the presence of diabetes, cancer, or self-reported stroke.

Physically active was defined as ≥5 d/wk of moderate or vigorous activity (>3METs).

Table 2 presents the results of the analyses on the association of food scores with depressive symptoms and dispositional optimism. These results show an inverse association between higher quintiles of the DHNaFS score and depressive symptoms (β = -0.108; P<0.001), besides a positive association between higher quintiles of the DHNaFS score and dispositional optimism (β = 0.074; P<0.001). Conversely, no associations were found between the DUNaFS score and depressive symptoms (β = -0.002; P = 0.93) or dispositional optimism (β = -0.014; P = 0.48).

**Table 2:** Quintiles of undesirable food score in relation to depressive symptoms and dispositional optimism in 2055 post-myocardial infarction patients.

**Dutch Healthy Nutrient and Food Score (DHNaFS)**	**Q1** **(n = 405)**	**Q2** **(n = 399)**	**Q3** **(n = 533)**	**Q4** **(n = 375)**	**Q5** **(n = 459)**	**Beta’s**	**P—value for trend**
Range in DHNaFS	2 to 16	17 to 20	21 to 24	25 to 27	28 to 38	-	-
Depressive symptoms:							
• Median (p25, p75)	2 (1,4)	2 (1,3)	1 (0,3)	1 (0,2)	1 (0,2)	-	<0.001*
• Crude	2.71 (0.14)	2.20 (0.12)	1.89 (0.09)	1.57 (0.09)	1.65 (0.09)	- 0.195	<0.001
• model 1	2.63 (0.11)	2.19 (0.11)	1.88 (0.10)	1.61 (0.11)	1.72 (0.10)	- 0.172	<0.001
• model 2	2.40 (0.10)	2.12 (0.10)	1.91 (0.09)	1.75 (0.10)	1.86 (0.09)	- 0.108	<0.001
Dispositional optimism:							
• Median (p25,p75)	6 (5,7)	6 (5,8)	7 (6,8)	7 (6,8)	7 (6,8)	-	<0.001*
• crude	5.94 (0.09)	6.15 (0.08)	6.39 (0.07)	6.36 (0.08)	6.63 (0.07)	0.154	<0.001
• model 1	6.01 (0.08)	6.15 (0.08)	6.41 (0.07)	6.33 (0.08)	6.58 (0.07)	0.126	<0.001
• model 2	6.15 (0.08)	6.19 (0.07)	6.39 (0.06)	6.24 (0.08)	6.49 (0.07)	0.074	<0.001
Dutch Undesirable Nutrient and Food Score (DUNaFS)	**Q1** (n = 413)	**Q2** (n = 485)	**Q3** (n = 346)	**Q4** (n = 533)	**Q5** (n = 394)		**P—value for trend**
Range in DUNaFS	5 to 19	20 to 24	25 to 27	28 to 32	33 to 49		
Depressive symptoms:							
• Median (p25,p75)	2 (0,3)	1 (0,3)	1 (0,2)	1 (0,3)	1 (0,3)	-	0.07*
• Crude	2.19 (0.13)	2.12 (0.12)	1.92 (0.13)	1.79 (0.09)	1.92 (0.11)	- 0.059	0.008
• model 1	2.06 (0.11)	2.09 (0.10)	1.92 (0.12)	1.87 (0.10)	2.04 (0.11)	- 0.019	0.37
• model 2	2.01 (0.10)	2.08 (0.09)	1.95 (0.11)	1.86 (0.09)	2.12 (0.10)	- 0.002	0.93
Dispositional optimism:							
• Median (p25,p75)	6 (5,8)	7 (5,8)	6 (6,8)	7 (6,8)	7 (5,8)	-	0.45*
• crude	6.19 (0.09)	6.31 (0.08)	6.34 (0.09)	6.39 (0.06)	6.33 (0.08)	0.043	0.05
• model 1	6.30 (0.08)	6.33 (0.07)	6.33 (0.08)	6.32 (0.07)	6.26 (0.08)	0.003	0.89
• model 2	6.33 (0.08)	6.33 (0.07)	6.32 (0.08)	6.32 (0.06)	6.21 (0.08)	- 0.014	0.48

Data are reported (adjusted) mean and standard errors (SE).

*p-values calculated with the non-parametric Jonckheere-Terpstra test.

Analysis of covariance (ANCOVA) was used to determine significance for linear trend over the quintiles of the diet (un)healthy score. Linear regression analysis was used to determine the beta’s. Depressive symptoms and dispositional optimism were introduced in the model as continuous variables.

Model 1: adjusted for age, sex.

Model 2: additionally adjusted for education, marital status, physical activity, body mass index, high alcohol use, current smoking, use of antidepressants, family history of depression, self-rated health, chronic disease, and treatment group.

In Table 3 we report the results of the univariate and multiple linear regression analyses on the association between the individual foods of the composite DHNaFS score and mental health. Depressive symptoms were inversely associated with higher consumption of vegetables (β = -0.074; P = 0.003), fruits (β = -0.047; P = 0.03), whole grains (β-0.090; P<0.001), fish (β = -0.051; P = 0.018), and low-fat dairy (β = -0.055; P = 0.009); whilst higher dispositional optimism was only associated with higher consumption of vegetables (β = 0.089; P = 0.001), whole grains (β = 0.087; P<0.001), fish (β = 0.043; P = 0.05), and low-fat dairy (β = 0.047; P = 0.03).

**Table 3:** Individual foods of the DHNaFS in relation to depressive symptoms and dispositional optimism at T2 in 2171 post-myocardial infarction patients.

	Depressive symptoms				Dispositional optimism			
	Crude Beta	P—value	Adjusted Beta*	P—value	Crude Beta	P—value	Adjusted Beta*	P—value
Vegetables	-0.120	<0.001	-0.074	0.003	0.108	<0.001	0.089	<0.001
Fruits	-0.070	0.001	-0.047	0.03	0.057	0.008	0.032	0.14
Whole grains	-0.106	<0.001	-0.090	<0.001	0.096	<0.001	0.087	<0.001
Potatoes	-0.092	<0.001	-0.041	0.09	0.045	0.036	-0.008	0.75
Protein enriched plants	-0.084	<0.001	-0.040	0.09	0.066	0.002	0.031	0.17
Lean meat	-0.060	0.005	-0.039	0.07	0.057	0.008	0.041	0.06
Eggs	-0.016	0.45	-0.006	0.77	0.025	0.24	0.019	0.39
Fish	-0.068	0.002	-0.051	0.018	0.062	0.004	0.043	0.05
Low-fat dairy	-0.067	0.002	-0.055	0.009	0.057	0.01	0.047	0.03
Oils and margarines	-0.016	0.45	0.013	0.54	-0.016	0.47	-0.040	0.07
Non-caloric drinks	-0.0002	0.93	0.027	0.21	0.017	0.43	-0.01	0.61

The Dutch Healthy Nutrient and Food Score (DHNaFS) included 11 nutrient-dense food groups: vegetables, fruit, whole grains, protein-rich plant foods (mostly legumes), potatoes, lean meat, fish, eggs, low-fat dairy (milk and yogurt), oils and soft margarines, and non-caloric drinks.

*: Adjusted beta’s by multiple regression analysis including all 11 food groups from the DHNaFS.

### Findings in relation to average food scores

The mean change of the healthy and undesirable food scores was 0.23 (SD = 5.70, P = 0.07) and -0.08 (SD = 6.15, P = 0.55), respectively, indicating that there was only a slight improvement in these healthy food scores between T1 and T2. The Pearson’s correlation coefficients were 0.55 (P<0.001) for the healthy food scores. There was an inverse association between average healthy food score over 41 months and depressive symptoms (-0.085; p<0.0001); whilst a positive association was found for dispositional optimism (0.084;p<0.001) (Table 4).

**Table 4:** Healthy food score over the preceding 41 months (T1 and T2) in relation to depressive symptoms and dispositional optimism at T2 in 2171 post-myocardial infarction patients.

Dutch Healthy Nutrient and Food Score (DHNaFS)	Q1 (n = 423)	Q2 (n = 433)	Q3 (n = 464)	Q4 (n = 401)	Q5 (n = 450)	Beta’s	P—value for trend
Range in mean DHNaFS	2 to 25	10 to 29	13 to 32	12 to 34	19 to 38	-	-
Depressive symptoms:							
• crude	2.83 (0.16)	2.02 (0.12)	1.75 (0.11)	1.77 (0.12)	1.60 (0.15)	-0.162	<0.001
• model 1	2.72 (0.16)	2.02 (0.12)	1.77 (0.11)	1.77 (0.12)	1.69 (0.15)	-0.139	<0.001
• model 2	2.52 (0.14)	1.91 (0.11)	1.84 (0.09)	1.90 (0.11)	1.81 (0.13)	-0.085	<0.001
Dispositional optimism:							
• crude	5.87 (0.12)	6.17 (0.08)	6.49 (0.08)	6.46 (0.09)	6.57 (0.11)	0.151	<0.001
• model 1	5.98 (0.11)	6.17 (0.08)	6.48 (0.07)	6.45 (0.08)	6.48 (0.11)	0.127	<0.001
• model 2	6.08 (0.11)	6.23 (0.08)	6.43 (0.07)	6.38 (0.08)	6.41 (0.10)	0.084	<0.001

Data are reported (adjusted) mean and standard errors (SE). Quintiles of the average (T2,T1)

Analysis of covariance (ANCOVA) was used to determine significance for linear trend over the quintiles of the diet healthy score. Linear regression analysis was used to determine the beta’s. Depressive symptoms and dispositional optimism were introduced in the model as continuous variables.

Model 1: adjusted for age, sex.

Model 2: additionally adjusted for healthy diet score at baseline, education, marital status, physical activity, body mass index. high alcohol use, current smoking, use of antidepressants, family history of depression, self-rated health, chronic disease, and treatment group.

## Discussion

Results from the present study suggest that a healthy dietary pattern is associated with better mental health in older patients with a history of cardiac infarction. Particularly, higher consumption of vegetables, fruit, whole grains, fish and low-fat dairy were associated with less depressive symptoms. Similar results were found for a healthy dietary pattern and higher levels of dispositional optimism, also for the individual foods. Furthermore, our results showed that average healthy food scores over 41 months of follow-up are also associated with better mental health outcomes. By contrast, no such associations were shown with the undesirable food scores.

Our finding of an association between a healthy dietary pattern and depressive symptoms is in line with the results of other observational studies that showed that high adherence to healthy dietary habits is associated with less depressive symptoms [14–17, 36]. In accordance with these previous studies, we also found that specifically the consumption of vegetables, fruits and whole grains is associated with lower risks of depression. We extend their findings by showing that these associations are also relevant in patients with a history of coronary heart disease and not only in community-dwelling populations.

Interestingly, our results showed that an undesirable diet was not independently associated with depressive symptoms. Therefore, our findings regarding undesirable diet are discrepant with some other studies that did find undesirable dietary patterns to predict depressive symptoms [37–39]. These conflicting results may be explained by methodological differences or the studied population. First of all, most studies were conducted among relatively young, healthy adults, whilst we studied older cardiovascular patients who were at increased risk for depressive symptoms. Secondly, other cohort studies used other (often short) dietary indices that have been constructed differently and are based to various national, nutritional guidelines that are similar but not completely concordant with the Dutch dietary guidelines. Finally, other studies have often assessed depression with self-report questions or have included the use of antidepressants as a proxy for the diagnosis of depression, whilst such medication may have been prescribed with other indications and depression may have remitted at the time of assessment.

Regarding the association between dietary patterns and optimism, our results were concordant with those of an earlier study among a younger, population-based sample [21, 22], post-menopausal women [24], women with fibromyalgia [25], and older men [23]. With this study, we add up to the emerging evidence on the association between dietary patterns and optimism in older men and women with a history of myocardial infarction.

A variety of biological and psychosocial mechanisms have been suggested to explain the association between diet and mental health. Some of the previously studied healthy dietary patterns, such as the Mediterranean, Norwegian, or Japanese diets, are anchored in social tradition. Mealtimes are events when the whole family comes together, and may thus serve as social gatherings, possibly enhancing mental health through enhancing intimacy and reducing loneliness [40]. It could be hypothesized that more healthy food choices are made when meals are consumed together [41]. From a biological point of view, various hypotheses have been formulated. A healthy diet may positively affect mood by increasing brain levels of monoamines [42], reduce apoptosis of the limbic system [43, 44], and have anti-inflammatory properties [45, 46]. The high content of antioxidants in fruits and vegetables is likely to be protective against the negative effects of oxidative stress at neuronal level, which has been associated with depression [47]. Particularly, the anti-inflammatory and anti-oxidant effects are likely to be of importance in preventing both cerebral changes associated with depression and chronic inflammatory states that influence coronary heart disease [48]. However, more (experimental) studies are needed to confirm this hypothesis.

A major strength of this study was the inclusion of a large, representative cohort of patients with coronary heart disease, and the assessment of the variables with well-validated scales. A specific focus on patients with coronary heart disease contributes to shed light on what factors might be important in the prevention and reduction of comorbidity in these patients. Furthermore, we differentiated between a healthy and undesirable food score, being able to show that mental health was more strongly associated with a healthy dietary patterns rather than with undesirable dietary patterns.

Nevertheless, some limitations should also be considered. Because dietary patterns were assessed using a self-report questionnaire, it is possible that the provided information resulted in a measurement error of actual food scores. However, the main limitation is inherent to the cross-sectional design of our study, as it hampers interpretation of the causal direction. Reverse causation cannot be excluded as it is also possible that depression influences eating habits by its intrinsic symptoms such as loss of appetite, disturbed sleep or craving to foods containing fat and sugar [49]. Furthermore, residual confounding may also have affected our results. Factors such as lower socioeconomic status and physical activity could influence both depression and dietary habits. It is plausible that patients who hold a healthier lifestyle, also engage in other protective lifestyle behaviors that prevent depression [50].

Our findings provide preliminary evidence of a relationship between dietary patterns and mental health in patients with coronary heart disease. Future research in controlled interventions and well powered prospective studies might help to elucidate the direction of these associations and establish whether cardiac patients may benefit from efforts to promote healthier diets, not only as a primary prevention of coronary heart disease, but also to reduce depressive symptoms and improve dispositional optimism [50, 51].

In conclusion, this study in participants with a history of MI showed that a healthy diet is associated with better mental health More research is needed to define the pathway of causality and to investigate the potential benefits of dietary interventions in improving mental health in cardiac patients.

## References

[1] Rutledge T, Reis VA, Linke SE, Greenberg BH, Mills PJ. Depression in heart failure a meta-analytic review of prevalence, intervention effects, and associations with clinical outcomes. J Am Coll Cardiol. 2006;48(8):1527–37. doi: 10.1016/j.jacc.2006.06.055 17045884 10.1016/j.jacc.2006.06.055

[2] Hackett ML, Pickles K. Part I: frequency of depression after stroke: an updated systematic review and meta-analysis of observational studies. Int J Stroke. 2014;9(8):1017–25. doi: 10.1111/ijs.12357 25117911 10.1111/ijs.12357

[3] Vos T, Flaxman AD, Naghavi M, Lozano R, Michaud C, Ezzati M, et al Years lived with disability (YLDs) for 1160 sequelae of 289 diseases and injuries 1990–2010: a systematic analysis for the Global Burden of Disease Study 2010. Lancet. 2012;380(9859):2163–96. doi: 10.1016/S0140-6736(12)61729-2 23245607 10.1016/S0140-6736(12)61729-2 PMC6350784

[4] Nicholson A, Kuper H, Hemingway H. Depression as an aetiologic and prognostic factor in coronary heart disease: a meta-analysis of 6362 events among 146 538 participants in 54 observational studies. Eur Heart J. 2006;27(23):2763–74. doi: 10.1093/eurheartj/ehl338 17082208 10.1093/eurheartj/ehl338

[5] Meijer A, Conradi HJ, Bos EH, Thombs BD, van Melle JP, de JP. Prognostic association of depression following myocardial infarction with mortality and cardiovascular events: a meta-analysis of 25 years of research. Gen Hosp Psychiatry. 2011;33(3):203–16. doi: 10.1016/j.genhosppsych.2011.02.007 21601716 10.1016/j.genhosppsych.2011.02.007

[6] Baumeister H, Hutter N, Bengel J. Psychological and pharmacological interventions for depression in patients with coronary artery disease. Cochrane Database Syst Rev. 2011;(9):CD008012 doi: 10.1002/14651858.CD008012.pub3 21901717 10.1002/14651858.CD008012.pub3 PMC7389312

[7] Casacalenda N, Perry JC, Looper K. Remission in major depressive disorder: a comparison of pharmacotherapy, psychotherapy, and control conditions. Am J Psychiatry. 2002;159(8):1354–60. doi: 10.1176/appi.ajp.159.8.1354 12153828 10.1176/appi.ajp.159.8.1354

[8] Papakostas GI, Ionescu DF. Towards new mechanisms: an update on therapeutics for treatment-resistant major depressive disorder. Mol Psychiatry. 2015;20(10):1142–50. doi: 10.1038/mp.2015.92 26148812 10.1038/mp.2015.92

[9] Pietrzak RH, Kinley J, Afifi TO, Enns MW, Fawcett J, Sareen J. Subsyndromal depression in the United States: prevalence, course, and risk for incident psychiatric outcomes. Psychol Med. 2013;43(7):1401–14. doi: 10.1017/S0033291712002309 23111093 10.1017/S0033291712002309

[10] Opie RS, O'Neil A, Itsiopoulos C, Jacka FN. The impact of whole-of-diet interventions on depression and anxiety: a systematic review of randomised controlled trials. Public Health Nutr. 2015;18(11):2074–93. doi: 10.1017/S1368980014002614 25465596 10.1017/S1368980014002614 PMC10271872

[11] Lai JS, Hiles S, Bisquera A, Hure AJ, McEvoy M, Attia J. A systematic review and meta-analysis of dietary patterns and depression in community-dwelling adults. Am J Clin Nutr. 2014;99(1):181–97. doi: 10.3945/ajcn.113.069880 24196402 10.3945/ajcn.113.069880

[12] Rahe C, Unrath M, Berger K. Dietary patterns and the risk of depression in adults: a systematic review of observational studies. Eur J Nutr. 2014;53(4):997–1013. doi: 10.1007/s00394-014-0652-9 24468939 10.1007/s00394-014-0652-9

[13] Sanhueza C, Ryan L, Foxcroft DR. Diet and the risk of unipolar depression in adults: systematic review of cohort studies. J Hum Nutr Diet. 2013;26(1):56–70. doi: 10.1111/j.1365-277X.2012.01283.x 23078460 10.1111/j.1365-277X.2012.01283.x

[14] Adjibade M, Assmann KE, Andreeva VA, Lemogne C, Hercberg S, Galan P, et al Prospective association between adherence to the Mediterranean diet and risk of depressive symptoms in the French SU.VI.MAX cohort. Eur J Nutr. 2017 Epub 2017/03/12. doi: 10.1007/s00394-017-1405-3 . 28283824 10.1007/s00394-017-1405-3

[15] Jacka FN, Cherbuin N, Anstey KJ, Butterworth P. Dietary patterns and depressive symptoms over time: examining the relationships with socioeconomic position, health behaviours and cardiovascular risk. PloS one. 2014;9(1):e87657 Epub 2014/02/04. doi: 10.1371/journal.pone.0087657 . 24489946 10.1371/journal.pone.0087657 PMC3906192

[16] McMartin SE, Jacka FN, Colman I. The association between fruit and vegetable consumption and mental health disorders: evidence from five waves of a national survey of Canadians. Preventive medicine. 2013;56(3–4):225–30. Epub 2013/01/09. doi: 10.1016/j.ypmed.2012.12.016 . 23295173 10.1016/j.ypmed.2012.12.016

[17] Tsai AC, Chang TL, Chi SH. Frequent consumption of vegetables predicts lower risk of depression in older Taiwanese—results of a prospective population-based study. Public Health Nutr. 2012;15(6):1087–92. Epub 2011/12/20. doi: 10.1017/S1368980011002977 . 22176686 10.1017/S1368980011002977

[18] Sanchez-Villegas A, Martinez-Gonzalez MA. Diet, a new target to prevent depression? BMC Med. 2013;11:3 doi: 10.1186/1741-7015-11-3 23286788 10.1186/1741-7015-11-3 PMC3570363

[19] Tinker LF, Rosal MC, Young AF, Perri MG, Patterson RE, Van HL, et al Predictors of dietary change and maintenance in the Women's Health Initiative Dietary Modification Trial. J Am Diet Assoc. 2007;107(7):1155–66. doi: 10.1016/j.jada.2007.04.010 17604744 10.1016/j.jada.2007.04.010

[20] Scheier MF, Carver CS, Bridges MW. Distinguishing optimism from neuroticism (and trait anxiety, self-mastery, and self-esteem): a reevaluation of the Life Orientation Test. Journal of personality and social psychology. 1994;67(6):1063–78. Epub 1994/12/01. . 7815302 10.1037//0022-3514.67.6.1063

[21] Serlachius A, Pulkki-Raback L, Elovainio M, Hintsanen M, Mikkila V, Laitinen TT, et al Is dispositional optimism or dispositional pessimism predictive of ideal cardiovascular health? The Young Finns Study. Psychol Health. 2015;30(10):1221–39. doi: 10.1080/08870446.2015.1041394 . 25985260 10.1080/08870446.2015.1041394

[22] Kelloniemi H, Ek E, Laitinen J. Optimism, dietary habits, body mass index and smoking among young Finnish adults. Appetite. 2005;45(2):169–76. Epub 2005/07/13. doi: 10.1016/j.appet.2005.05.001 . 16009454 10.1016/j.appet.2005.05.001

[23] Giltay EJ, Geleijnse JM, Zitman FG, Buijsse B, Kromhout D. Lifestyle and dietary correlates of dispositional optimism in men: The Zutphen Elderly Study. J Psychosom Res. 2007;63(5):483–90. doi: 10.1016/j.jpsychores.2007.07.014 17980220 10.1016/j.jpsychores.2007.07.014

[24] Hingle MD, Wertheim BC, Tindle HA, Tinker L, Seguin RA, Rosal MC, et al Optimism and diet quality in the Women's Health Initiative. J Acad Nutr Diet. 2014;114(7):1036–45. doi: 10.1016/j.jand.2013.12.018 24556429 10.1016/j.jand.2013.12.018 PMC4071123

[25] Ruiz-Cabello P, Soriano-Maldonado A, Delgado-Fernandez M, Alvarez-Gallardo IC, Segura-Jimenez V, Estevez-Lopez F, et al Association of Dietary Habits with Psychosocial Outcomes in Women with Fibromyalgia: The al-Andalus Project. Journal of the Academy of Nutrition and Dietetics. 2017;117(3):422–32.e1. Epub 2016/11/29. doi: 10.1016/j.jand.2016.09.023 . 27890478 10.1016/j.jand.2016.09.023

[26] Kromhout D, Giltay EJ, Geleijnse JM. n-3 fatty acids and cardiovascular events after myocardial infarction. The New England journal of medicine. 2010;363(21):2015–26. Epub 2010/10/12. doi: 10.1056/NEJMoa1003603 . 20929341 10.1056/NEJMoa1003603

[27] Geleijnse JM, Giltay EJ, Schouten EG, de Goede J, Oude Griep LM, Teitsma-Jansen AM, et al Effect of low doses of n-3 fatty acids on cardiovascular diseases in 4,837 post-myocardial infarction patients: design and baseline characteristics of the Alpha Omega Trial. American heart journal. 2010;159(4):539–46.e2. Epub 2010/04/07. doi: 10.1016/j.ahj.2009.12.033 . 20362710 10.1016/j.ahj.2009.12.033

[28] Feunekes GI, Van Staveren WA, De Vries JH, Burema J, Hautvast JG. Relative and biomarker-based validity of a food-frequency questionnaire estimating intake of fats and cholesterol. Am J Clin Nutr. 1993;58(4):489–96. Epub 1993/10/01. . 8379504 10.1093/ajcn/58.4.489

[29] Feunekes IJ, Van Staveren WA, Graveland F, De Vos J, Burema J. Reproducibility of a semiquantitative food frequency questionnaire to assess the intake of fats and cholesterol in The Netherlands. International journal of food sciences and nutrition. 1995;46(2):117–23. Epub 1995/05/01. . 7621083 10.3109/09637489509012539

[30] NEVO-tabel: Nederlands Voedingsstoffenbestand 2006/Stichting Nederlands Voedingsstoffenbestand [Internet]. 2006.

[31] Sijtsma FP, Soedamah-Muthu SS, de GJ, Oude Griep LM, Geleijnse JM, Giltay EJ, et al Healthy eating and lower mortality risk in a large cohort of cardiac patients who received state-of-the-art drug treatment. Am J Clin Nutr. 2015 doi: 10.3945/ajcn.115.112276 26490494 10.3945/ajcn.115.112276 PMC4658460

[32] de Craen AJ, Heeren TJ, Gussekloo J. Accuracy of the 15-item geriatric depression scale (GDS-15) in a community sample of the oldest old. International journal of geriatric psychiatry. 2003;18(1):63–6. Epub 2002/12/24. doi: 10.1002/gps.773 . 12497557 10.1002/gps.773

[33] Yesavage JA, Brink TL, Rose TL, Lum O, Huang V, Adey M, et al Development and validation of a geriatric depression screening scale: a preliminary report. Journal of psychiatric research. 1982;17(1):37–49. Epub 1982/01/01. . 7183759 10.1016/0022-3956(82)90033-4

[34] Giltay EJ, Kamphuis MH, Kalmijn S, Zitman FG, Kromhout D. Dispositional optimism and the risk of cardiovascular death: the Zutphen Elderly Study. Archives of internal medicine. 2006;166(4):431–6. Epub 2006/03/01. doi: 10.1001/archinte.166.4.431 . 16505263 10.1001/archinte.166.4.431

[35] Schuit AJ, Schouten EG, Westerterp KR, Saris WH. Validity of the Physical Activity Scale for the Elderly (PASE): according to energy expenditure assessed by the doubly labeled water method. Journal of clinical epidemiology. 1997;50(5):541–6. Epub 1997/05/01. . 9180646 10.1016/s0895-4356(97)00010-3

[36] Lai JS, Oldmeadow C, Hure AJ, McEvoy M, Byles J, Attia J. Longitudinal diet quality is not associated with depressive symptoms in a cohort of middle-aged Australian women. Br J Nutr. 2016;115(5):842–50. doi: 10.1017/S000711451500519X 26787123 10.1017/S000711451500519X

[37] Akbaraly TN, Sabia S, Shipley MJ, Batty GD, Kivimaki M. Adherence to healthy dietary guidelines and future depressive symptoms: evidence for sex differentials in the Whitehall II study. Am J Clin Nutr. 2013;97(2):419–27. doi: 10.3945/ajcn.112.041582 23283506 10.3945/ajcn.112.041582 PMC3545684

[38] Le Port A, Gueguen A, Kesse-Guyot E, Melchior M, Lemogne C, Nabi H, et al Association between dietary patterns and depressive symptoms over time: a 10-year follow-up study of the GAZEL cohort. PloS one. 2012;7(12):e51593 Epub 2012/12/20. doi: 10.1371/journal.pone.0051593 . 23251585 10.1371/journal.pone.0051593 PMC3520961

[39] Lucas M, Chocano-Bedoya P, Schulze MB, Mirzaei F, O'Reilly EJ, Okereke OI, et al Inflammatory dietary pattern and risk of depression among women. Brain, behavior, and immunity. 2014;36:46–53. Epub 2013/10/08. doi: 10.1016/j.bbi.2013.09.014 . 24095894 10.1016/j.bbi.2013.09.014 PMC3947176

[40] Zaborskis A, Zemaitiene N, Borup I, Kuntsche E, Moreno C. Family joint activities in a cross-national perspective. BMC public health. 2007;7:94 Epub 2007/06/01. doi: 10.1186/1471-2458-7-94 . 17537247 10.1186/1471-2458-7-94 PMC1904207

[41] Shatenstein B, Gauvin L, Keller H, Richard L, Gaudreau P, Giroux F, et al Individual and collective factors predicting change in diet quality over 3 years in a subset of older men and women from the NuAge cohort. Eur J Nutr. 2016;55(4):1671–81. Epub 2015/07/15. doi: 10.1007/s00394-015-0986-y . 26169872 10.1007/s00394-015-0986-y

[42] Sarris J, Stough C, Bousman C, Murphy J, Savage K, Smith DJ, et al An adjunctive antidepressant nutraceutical combination in treating major depression: Study protocol, and clinical considerations. Advances in Integrative Medicine. 2015;2(1):49–55.

[43] Rondeau I, Picard S, Bah TM, Roy L, Godbout R, Rousseau G. Effects of different dietary omega-6/3 polyunsaturated fatty acids ratios on infarct size and the limbic system after myocardial infarction. Can J Physiol Pharmacol. 2011;89(3):169–76. doi: 10.1139/Y11-007 21423290 10.1139/Y11-007

[44] Gilbert K, Arseneault-Breard J, Flores MF, Beaudoin A, Bah TM, Tompkins TA, et al Attenuation of post-myocardial infarction depression in rats by n-3 fatty acids or probiotics starting after the onset of reperfusion. Br J Nutr. 2013;109(1):50–6. doi: 10.1017/S0007114512003807 23068715 10.1017/S0007114512003807

[45] Sanchez-Villegas A, Ruiz-Canela M, Fuente-Arrillaga C, Gea A, Shivappa N, Hebert JR, et al Dietary inflammatory index, cardiometabolic conditions and depression in the Seguimiento Universidad de Navarra cohort study. Br J Nutr. 2015;114(9):1471–9. doi: 10.1017/S0007114515003074 26344165 10.1017/S0007114515003074

[46] Hastings CN, Sheridan H, Pariante CM, Mondelli V. Does Diet Matter? The Use of Polyunsaturated Fatty Acids (PUFAs) and Other Dietary Supplements in Inflammation-Associated Depression. Curr Top Behav Neurosci. 2016 doi: 10.1007/7854_2016_31 10.1007/7854_2016_31 27431396

[47] Liu X, Yan Y, Li F, Zhang D. Fruit and vegetable consumption and the risk of depression: A meta-analysis. Nutrition (Burbank, Los Angeles County, Calif). 2016;32(3):296–302. Epub 2015/12/23. doi: 10.1016/j.nut.2015.09.009 . 26691768 10.1016/j.nut.2015.09.009

[48] Martinez-Gonzalez MA, Sanchez-Villegas A. Food patterns and the prevention of depression. The Proceedings of the Nutrition Society. 2016;75(2):139–46. Epub 2016/02/24. doi: 10.1017/S0029665116000045 . 26898781 10.1017/S0029665116000045

[49] Whitaker KM, Sharpe PA, Wilcox S, Hutto BE. Depressive symptoms are associated with dietary intake but not physical activity among overweight and obese women from disadvantaged neighborhoods. Nutr Res. 2014;34(4):294–301. doi: 10.1016/j.nutres.2014.01.007 24774065 10.1016/j.nutres.2014.01.007 PMC4004962

[50] Whooley MA, de Jonge P, Vittinghoff E, Otte C, Moos R, Carney RM, et al Depressive symptoms, health behaviors, and risk of cardiovascular events in patients with coronary heart disease. Jama. 2008;300(20):2379–88. Epub 2008/11/27. doi: 10.1001/jama.2008.711 . 19033588 10.1001/jama.2008.711 PMC2677371

[51] Hu FB, Willett WC. Optimal diets for prevention of coronary heart disease. Jama. 2002;288(20):2569–78. Epub 2002/11/28. . 12444864 10.1001/jama.288.20.2569

